# Immunotherapeutic strategies targeting B cell maturation antigen in multiple myeloma

**DOI:** 10.1186/s40779-021-00302-x

**Published:** 2021-01-27

**Authors:** Yi Fang, Jian Hou

**Affiliations:** Department of Hematology, Renji Hospital, School of Medicine, Shanghai Jiao Tong University, 160 Pujian Road, Shanghai, 200127 China

**Keywords:** B cell maturation antigen, Multiple myeloma, Vaccine, Antibody, CAR T-cells

## Abstract

Multiple myeloma (MM) is the second most common hematologic malignancy, and is characterized by the clonal expansion of malignant plasma cells. Despite the recent improvement in patient outcome due to the use of novel therapeutic agents and stem cell transplantation, all patients eventually relapse due to clone evolution. B cell maturation antigen (BCMA) is highly expressed in and specific for MM cells, and has been implicated in the pathogenesis as well as treatment development for MM. In this review, we will summarize representative anti-BCMA immune therapeutic strategies, including BCMA-targeted vaccines, anti-BCMA antibodies and BCMA-targeted CAR cells. Combination of different immunotherapeutic strategies of targeting BCMA, multi-target immune therapeutic strategies, and adding immune modulatory agents to normalize anti-MM immune system in minimal residual disease (MRD) negative patients, will also be discussed.

## Introduction

Multiple myeloma (MM) is the second most common hematologic malignancy, and accounts for 10% of all malignant hematologic diseases [1]. MM is characterized by the expansion of malignant plasma cells (PC) in the bone marrow. These clonal plasma cells produce excessive monoclonal immunoglobulin (M protein), leading to hypercalcemia, renal failure, anemia and bone lesions (CRAB). During the last decade, patient outcome has been significantly improved in both newly diagnosed MM (NDMM) and relapsed and refractory MM (RRMM) patients due to the use of novel therapeutic agents, such as proteasome inhibitors (PIs), immunomodulatory drugs (IMiDs) and monoclonal antibodies (MAbs) targeting CD38 or CS-1/SLAMF7 [2, 3], as well as autologous stem cell transplantation (ASCT) [4]. Despite these advances, all patients eventually relapse, even in patients without minimal residual disease (MRD), due to clone evolution that evades cytotoxicity by therapeutic agents [5].

B cell maturation antigen (BCMA) is one of the most specific and highly expressed antigens of MM [6]. Treatments targeting BCMA represent promising pipelines to develop novel effective therapies for MM [7]. In the following chapters, we will cover BCMA-targeted vaccines, anti-BCMA antibodies and BCMA-targeted CAR cells [7].

## BCMA

**Fig. 1:**
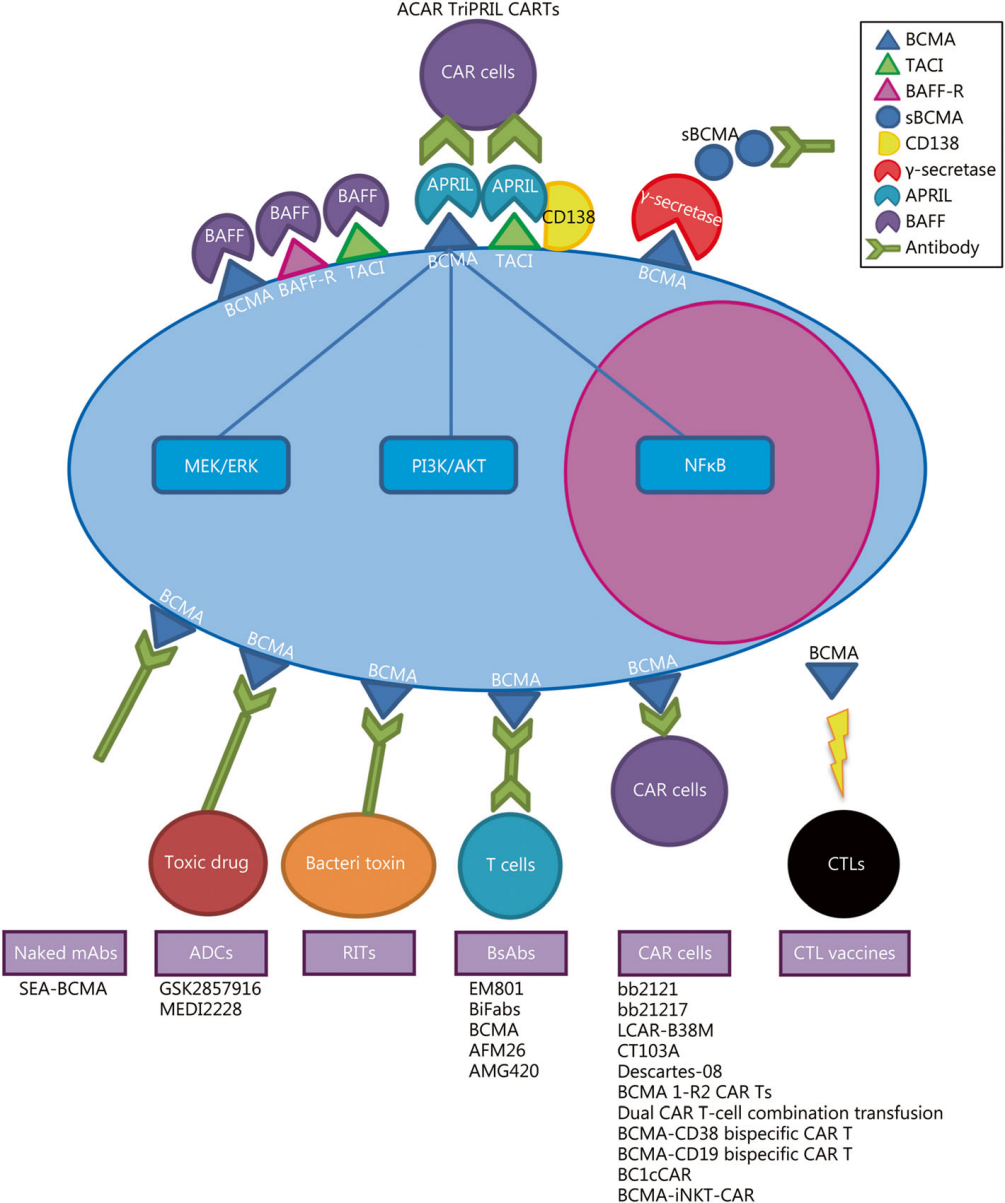
The schematic diagram of BCMA mediated signal conduction and immunotherapeutic strategies targeting BCMA in MM. BCMA is one of the most specific antigens in MM, which is closely related to transmembrane protein BAFF-R and TACI. BAFF binds to BCMA, BAFF-R and TACI, while APRIL binds to BCMA and TACI depending on CD138 (syndecan-1). BCMA delivers critical signal via APRIL, which regulates MEK/ERK, PI3K/AKT and NFκB pathway, to induce B-cell maturation and differentiation into plasma cells. BCMA is a natural substrate for γ-secretase that forms sBCMA, which may neutralize anti-BCMA immune drug. Immune therapeutic strategies targeting BCMA include BCMA-targeted vaccines, anti-BCMA antibodies (such as ADCs, RITs and BsAbs) and BCMA-targeted CAR cells including autologous or allogeneic BCMA CAR T cells, dual-antigen targeting CAR T-cell strategies and BCMA CAR on other cells. Representative therapies discussed in the review are listed. BCMA: B cell maturation antigen; APRIL: A proliferation-inducing ligand; BAFF: B-cell activating factor; BAFF-R: B-cell activating factor receptor; TACI: Transmembrane activator and calcium modulator and cyclophilin ligand interactor; sBCMA: Soluble BCMA; mAbs: Monoclonal antibodies; ADCs: Monoclonal antibodies bound to toxic drug; RITs: Recombinant immunotoxins; BsAbs: Bispecific antibodies; CAR: Chimeric antigen receptor; CTL: Cytotoxic T-cell

BCMA, also referred to as TNFRS17 or CD269, is a type III transmembrane glycoprotein in the tumor necrosis factor receptors (TNFR) superfamily. It contains cysteine-rich extracellular domains, and is selectively expressed in late memory B cells committed to PC differentiation. BCMA is expressed on plasmablasts and differentiated PCs exclusively, and is absent on naïve and most memory B cells. Therefore, BCMA is required for the differentiation and survival of PCs, but they may not be critical for overall B-cell homeostasis [8]. BCMA is activated by either proliferation-inducing ligand (APRIL) or B-cell activating factor (BAFF), and regulates B-cell maturation and differentiation into plasma cells. BCMA is also closely related to two other functional type III transmembrane proteins: BAFF receptor (BAFF-R) and transmembrane activator and calcium modulator and cyclophilin ligand interactor (TACI). BAFF binds to BCMA, BAFF-R and TACI, whereas APRIL binds to BCMA and TACI depending on heparin sulfate proteoglycan (CD138/syndecan-1), suggesting a more PC-specific role of APRIL. APRIL also has higher affinity than BAFF for BCMA [9]. Overall, BCMA delivers critical signal via APRIL to regulate key signalling pathways, such as MEK/ERK, PI3K/AKT and NF-κB pathway, and ultimately induces immunoglobulin isotype switching and survival of plasmablasts and PCs in the bone marrow [10] (Fig. 1).

BCMA is one of the most selectively expressed cell surface receptors on MM cell lines and primary myeloma cells, but undetectable on normal human tissues [11]. BCMA expression is increased with progression from monoclonal gammopathy of undetermined significance to asymptomatic MM and then symptomatic MM [12]. On the contrary, BAFF-R and TACI are hardly detectable or present at significantly lower level compared with BCMA in MM cells [8]. APRIL/BCMA axis is constitutively activated in MM [8].

BCMA is a natural substrate for γ-secretase that reduces membrane BCMA level and forms soluble BCMA (sBCMA), with the result of decreased binding of membrane BCMA to APRIL and BAFF [13]. Serum sBCMA is elevated in MM patients and predicts the clinical outcome, and thus is regarded as a biomarker to monitor disease progression [14]. sBCMA could neutralize anti-BCMA immune drugs, and thus may interfere with anti-BCMA immune therapy [15].

## BCMA-targeted immunotherapeutic strategies in MM

BCMA is a specific tumor-associated target antigen (TAA) for MM. Immune therapeutic strategies targeting BCMA under active development include BCMA-targeted vaccines, anti-BCMA antibodies and anti-BCMA CAR cells (Fig. 1).

### BCMA-targeted vaccines

BCMA-targeted vaccines under development include BCMA-mRNA-loaded dendritic cell (DC) and BCMA-peptide specific cytotoxic T-cell (CTL) vaccines.

#### BCMA-mRNA-loaded DC vaccines

TAA-mRNA-loaded DC vaccination has been tested in a clinical trial in MM patients in stable remission after high-dose melphalan and ASCT [16]. Briefly, mature monocyte-derived DCs were pulsed with keyhole limpet hemocyanin and electroporated with mRNA of MAGE3, survivin and BCMA, respectively. Twelve MM patients were vaccinated three times with all three types of TAA-mRNA-loaded autologous DCs via intravenous and intradermal injection at biweekly intervals. TAA-specific T-cells were detected in only two patients. One patient had MAGE3-specific CD4^+^ and CD8^+^ T-cells, and CD3^+^ T-cells against BCMA and survivin. This patient received additional therapy at 10 months after vaccination due to progressing disease (PD). The other patient had low numbers of MAGE3 and BCMA-reactive CD8^+^ T-cells, and had stable M-protein levels for 38 months, but developed prostate cancer 30 months after the first DC vaccination. These findings demonstrated that these BCMA-mRNA-loaded DCs are well tolerated with limited toxicity, but the capability of inducing BCMA-T-cell responses is minimal. No subsequent studies have been reported, indicating a major need to increase anti-MM efficacy.

#### BCMA-peptide specific CTL vaccines

In BCMA-peptide specific CTL vaccines, the choice of BCMA peptides is critically important. The native BCMA72–80 (VLMFLLRKI) and BCMA54–62 (AILWTCLGL) peptides had the highest HLA-A2 binding affinity, which were chosen for engineered modification for further improvement of HLA-A2 affinity [17]. The engineered heteroclitic BCMA72–80 and BCMA54–62 display better affinity and stability than their native peptides, and induce highly functional BCMA-specific CTLs with increased activation and co-stimulatory molecule expression. Notably, The heteroclitic BCMA72–80 specific CTLs demonstrate poly-functional Th1-specific activities against MM, correlated with expansion of Tetramer^+^ and memory CD8^+^ CTLs. Central memory CD8^+^ T-cells specific to heteroclitic BCMA72–80 demonstrate the greatest anti-MM activities, and increase immune function when treated with anti-OX40 or anti-LAG-3. Also, the CTL vaccine was prepared from multiple HLA-A2^+^ normal donors and effective against MM cells from HLA-A2^+^ patients, and thus could be used with less MHC restriction, although HLA-A2 negative MM patients cannot be covered.

### Anti-BCMA antibodies

A variety of strategies based on anti-BCMA antibody have been developed in recent years. These include naked anti-BCMA mAbs, mAbs bound to toxic drug (antibody-drug conjugates, ADCs) or bacterial toxin (recombinant immunotoxin, RITs), and bispecific antibodies (BsAbs) that target both BCMA-expressing MM cells and CD3-expressing T-cells. Anti-BCMA antibodies are capable of inducing cytotoxic activity in MM cells without antigen-presenting cells and co-stimulatory molecules, and with no MHC restriction.

#### Naked BCMA mAbs

SG1 is a naked anti-BCMA mAb that blocks APRIL–dependent activation of NF-κB in a dose-dependent manner in vitro [18]. SG1 promotes cytotoxicity of MM cell lines after chimerization with and without Fc mutations. The Fc mutations enhance FcγRIIIA binding and increase antibody-dependent cell-mediated cytotoxicity (ADCC) against MM cells. SG1 also induces direct cytotoxic activity by conjugation to the cytotoxic drug monomethyl auristatin F (MMAF). Therefore, SG1 shows cytotoxic activity both as naked IgG and as ADC, but no further study has been conducted to evaluate the role of SG1 alone or as ADC in MM patients.

SEA-BCMA is a humanized afucosylated IgG1 anti-BCMA antibody that enhances FcγRIII binding through SEA technology. It increases ADCC and antibody-dependent cellular phagocytosis (ADCP) potency against MM cells and blocks the signal transduction from BCMA [19]. SEA-BCMA induced potent anti-MM activity in all seven xenograft models tested. With repeated dosing, it induced durable regression and prolonged survival. SEA-BCMA also targeted MM cells in the presence of sBCMA. SEA-BCMA was well-tolerated in vitro as well as in cynomolgus monkeys. A clinical trial using SEA-BCMA as monotherapy for MM is currently ongoing (NCT03582033).

#### Anti-BCMA ADCs

Anti-BCMA ADCs are anti-BCMA mAbs conjugated to cytotoxic agents. GSK2857916, also referred to as J6M0-mcMMAF, is a humanised afucosylated IgG1 monoclonal ADC. An anti-BCMA mAb (J6M0) was conjugated to tubulin polymerisation inhibitor MMAF by a protease-resistant maleimidocaproyl linker. Upon binding to the surface of BCMA-expressing cells, GSK2857916 is rapidly internalised and transported to the lysosome where J6M0 is degraded and the active cytotoxic cys-mcMMAF is released. Afucosylation of the anti-BCMA mAb enhances the binding to FcγRIIIa and the recruitment and activation of immune effector cells. GSK2857916 demonstrated selective and potent anti-MM activities by multiple cytotoxic mechanisms against MM cell lines, primary MM patient myeloma cells, and subcutaneous and disseminated MM mouse models [20, 21]. GSK2857916 arrests cell cycle at G_2_/M phase, induces caspase 3-depedent apoptosis, inhibits colony formation and increases ADCC potency against MM cells. The apoptotic and ADCC activity was further enhanced by lenalidomide. GSK2857916 also recruits macrophages and mediates antibody-dependent cellular phagocytosis (ADCP) of MM cells [20]. GSK2857916 monotherapy has been evaluated in a multi-center and open-label phase 1 study in RRMM patients (NCT02064387) [22]. After the dose-escalation part (38 patients), 3.40 mg/kg of GSK2857916 every 3 weeks was chosen for subsequent dose expansion (35 patients). Corneal events, the most common adverse events (AE), were mostly grade 1 or 2 and resulted in two treatment discontinuations in dose-escalation and no discontinuations in dose expansion part. The most common grade 3 or 4 events were thrombocytopenia and anemia. There were 12 treatment-related serious adverse events (SAEs) and no treatment-related deaths. The overall response rate (ORR) in dose expansion part was 60% (21/35; 18 patients with ≥ very good partial response (VGPR). The median progression-free survival (PFS) was 7.9 months.

MEDI2228 is an anti-BCMA ADC that conjugated anti-BCMA mAb to synthetic pyrrolobenzodiazepine (PBD) dimer tesirine via a cleavable valine-alanine dipeptide linker, with an average drug-to-antibody ratio of 2 and loss of binding to FcγR [23]. MEDI2228 is rapidly internalized and trafficked to lysosomes where the PBD is released, causing cell death via accumulation of DNA damage. MEDI2228 targets both MM cells and myeloma progenitor cells (MPCs). Notably, MEDI2228 is more cytotoxic to MPCs in the head-to-head comparison with anti-BCMA ADC conjugated with microtubule inhibitor MMAF [23]. Furthermore, critical DNA damage response (DDR) could be activated by MEDI2228 via phosphorylation of ATM/ATR kinases, checkpoint kinases (CHK)1/2 and H2AX, but not by MMAF ADC. In cutaneous human MM xenograft mouse models, MEDI2228 inhibits tumor growth, and may even results in complete tumor regression [23]. In a study by Tai et al., MEDI2228 acts synergistically with bortezomib to induce apoptosis of MM cell lines as well as significant tumor necrosis in mice. MEDI2228 also synergizes with DDR checkpoint inhibitors to enhance MM cell cytotoxicity [24]. In addition, MEDI2228 demonstrates the preferential binding of membrane-bound BCMA in the presence of sBCMA [23]. A clinical trial of MEDI2228 is currently ongoing as monotherapy for MM (NCT03489525).

#### Anti-BCMA RITs

Anti-BCMA RITs are chimeric proteins consisting of Fab or Fv portion of a BCMA antibody and Pseudomonas exotoxin A (PE). The anti-BCMA RIT LMB-70 (BM306-Fab-LRggs) contains the Fab portion of a BCMA mAb (BM306) fused to domain III of PE. It has high cytotoxic activity against BCMA expressing cell lines and primary myeloma cells from MM patients and decreases the volume of subcutaneous myeloma in xenograft mice but does not induce complete responses [25]. Disulfide-stabilized (ds) Fv-RIT has advantages over Fab-RIT due to higher expression and refolding efficiency. Three anti-BCMA RITs have been produced using Fvs from three different anti-BCMA antibodies. All three possess cytotoxic activities in H929 cells, with LMB-75 (BM306-dsFv-LRggs) having the most potent activity. Fusing these anti-BCMA RITs with albumin-binding domains (ABDs) result in decreased cytotoxic activity and much longer half-life in mouse, albeit still significantly shorter than other published ABD-fusion RITs [26].

#### Anti-BCMA BsAb

Anti-BCMA BsAbs contain two domains, one binding to CD3ε on T-cells and the other to BCMA on MM cells. Due to this design, anti-BCMA BsAbs selectively direct the cytolytic activity of T-cells to MM cells. There are two types of BsAbs: 1) IgG-like molecules that retain Fc-mediated functions, such as serum stability, ADCC, complement-dependent cytotoxicity (CDC), and ADCP; 2) non-IgG-like molecules with smaller size that enhances tissue penetration but with shorter half-life. Non-IgG-like molecules also have reduced non-specific activation of innate immune cells due to lack of Fc region [27].

##### IgG-like anti-BCMA BsAb

EM801 is an IgG-based BCMA-T-cell BsAb crosslinking CD3^+^ T-cells and myeloma cells. To minimize unspecific T-cell activation, it is constructed as an asymmetric two-arm IgG1-based human antibody, binding bivalently to BCMA with a high affinity and monovalently to CD3εwith a low affinity. EM801 activates CD4^+^/CD8^+^ T-cells and induces secretion of interferon-γ, granzyme B, and perforin. At nanomolar concentrations, EM801 induced killing of myeloma cells in 24 of 31 (77%) NDMM patients and 10 of 12 (83%) RRMM patients. In myeloma xenograft mouse model, EM801 induced tumor regression in six of nine mice. EM801 depleted BCMA^+^ cells in cynomolgus monkey [28]. These findings suggest that EM801 is potent anti-BCMA drug candidate for MM treatment.

Anti-BCMA bispecific Fab molecules (BiFabs BCMA) activated T-cells in vitro and induced rapid tumor regression in orthotopic xenograft model of MM. Interestingly, BiFabs BCMA activated T-cells to kill MM cells and induced 20-fold more potent killing of MM cell lines than anti-CD3 × anti-CS-1 (BiFab-CS1), possibly due to the affinity to target and/or epitope location [26]. The cytotoxic activities of BiFab-BCMA are comparable to those of anti-BCMA CAR-T both in vitro and in vivo, indicating promising treatment option for MM [29].

AFM26 is a tetravalent bispecific tandem diabody (TandAb) that crosslinks myeloma cells and NK cells by specifically targeting BCMA and CD16A. The affinity-improved anti-CD16A domain makes AFM26 bivalently bind to NK cells, resulting in high avidity and prolonged cell surface retention in the presence of IgG. In preclinical studies, AFM26 potently induced NK-cell-mediated lysis of MM cells expressing low levels of BCMA at low effector: target ratios, even in presence of polyclonal IgG, suggesting that AFM26 exerts ADCC activity at low antibody concentrations in presence of IgG despite the presence of serum IgG or IgG M-protein in about half of MM patients [30].

##### Non-IgG-like anti-BCMA BsAb

Anti-BCMA bispecific T-cell engager (BiTE) belongs to non-IgG-like molecules, and contains two single-chain variable fragments (scFvs), one binding to CD3ε molecules on T-cells and the other to BCMA on MM cells. Anti-BCMA BiTEs can activate T-cell to direct the cytolytic activity to MM cells at sub-picomolar concentrations.

AMG 420, also known as BI 836909, is a BiTE that binds monovalently to BCMA and CD3ε. AMG 420 crosslinks both BCMA^+^ MM cells and CD3^+^ T-cells, and forms a cytolytic synapse to activate CD3^+^ T-cells and lyses BCMA^+^ MM cells [31]. In in vitro assays, AMG 420 can activate T-cells, release cytokines and induce T-cell proliferation and selectively induce lysis of MM cells expressing BCMA. In ex vivo assays, AMG 420 induced MM cell lysis in primary MM cells from both NDMM and RRMM patients. AMG-420 depleted MM cells in a subcutaneous NCI-H929 xenograft model and prolonged survival in an orthotopic L-363 xenograft mouse model. AMG 420 depleted BCMA^+^ plasma cells in the bone marrow of cynomolgus monkey. In addition, AMG 420 is not influenced by the presence of bone marrow stromal cells, sBCMA and APRIL. A first-in-human (FIH) phase I dose escalation study of continuous intravenous doses of AMG 420 was conducted in RRMM patients who progressed after ≥2 lines of therapy including PIs and IMiDs (NCT02514239) [32]. Forty two patients received AMG 420 at 0.2–800 μg/d. Twenty patients (48%) developed SAEs (infections, polyneuropathy and edema). There were no grade ≥ 3 CNS toxicities. There were 2 deaths from AEs but none was related to treatment. No anti-AMG 420 Ab were detected. ORR was 31% (13/42) with 6 sCRs, 3 CRs, 2 VGPRs and 2 PRs. At maximum tolerated dose (MTD) of 400 μg/d, 70% of patients (7/10) responded, including 5 (50%) MRD-negative sCRs, 1 VGPR, and 1 PR, with all 7 patients responding in the first cycle and some responses lasted over a year. A phase 1b/2 multicenter, open-label expansion trial (NCT03836053) in RRMM patients is ongoing to assess the safety and efficacy of 400 μg/d AMG 420 as monotherapy.

AMG 701 is an anti-BCMA BiTE with extended half-life. It induced T-cell-dependent cellular cytotoxicity (TDCC) of BCMA^+^ MM cells, and caused T-cell changes in CD107a degranulation, IFNγ and TNFα production, and CD8^+^ and CD4^+^ T-cell proliferation. Combination of AMG 701 and lenalidomide induced remarkably higher anti-MM efficacy than either monotherapy. Currently, a phase I trial of AMG 701 as monotherapy (NCT03287908) is ongoing in RRMM patients [33].

NanoMuTE is a nanoparticle BiTE (nano BiTE), in which more than three total antibodies are conjugated to the surface of a liposome, one targeting T-cells and the other two targeting different epitopes on MM cells. The nanoparticle multi-specific T-cell engager was designed by conjugating the MoAb against BCMA, CS1 and CD38, together with anti-CD3. Compared with CD38/CD3, BCMA/CD3, or CS1/CD3 nano BiTE, the BCMA/CS1/CD38/CD3 NanoMuTEs had significantly higher binding ability to MM cell lines and primary patient MM cells and induced greater activation of T-cells and T-cell-redirected MM cell lysis. The NanoMuTEs and nano BiTE redirected T-cells to the tumor site and reduced tumor burden in MM-bearing mice [34].

### CAR strategies targeting BCMA

In the past 5 years, CAR strategies targeting BCMA, especially BCMA-targeted CAR T-cells, have been evaluated as a significant milestone in adoptive cellular therapy for MM. These include autologous or allogeneic BCMA CAR T-cells, dual-antigen targeting CAR T-cells and BCMA CAR on other cells.

#### BCMA CAR T-cells

BCMA CAR contains a BCMA recognition domain, an intracellular signalling domain such as CD3ζ, and a costimulatory domain such as CD28 and 4-1BB. CAR is packed into modified lentiviral or retroviral vectors to transfect autologous or allogeneic T-cells collected via leukapheresis from peripheral blood of the patients or donors. These CAR T-cells were expanded ex vivo for 2 to 3 weeks, and then infused into the patients after lymphodepleting chemotherapy. CAR T-cells can recognize antigen only on the cell surface in a MHC-independent manner.

##### Autologous BCMA CAR T-cells

bb2121 are BCMA CAR T-cells produced by transducing autologous T-cells via a lentiviral vector BB2121 with a second-generation CAR incorporating an anti-BCMA scFv, a CD3 ζ signaling domain and a 4-1BB co-stimulatory domain. bb2121 rapidly and sustainably eliminated MM cells and resulted in 100% survival after single-dose administration in a MM mouse model [35]. An open-label, phase 1 study of bb2121 (NCT02658929) has been conducted in 33 RRMM patients who received at least three previous lines of therapy including PIs and IMiDs or were refractory to both drugs, with the data-cutoff date of 6.2 months after the last infusion date [36]. Hematologic AEs were the most common events, and included grade 3 or higher neutropenia, leukopenia, anemia, and thrombocytopenia. 25 of 33 patients (76%) had cytokine release syndrome (CRS) with 70% at grade 1 or 2 and 6% at grade 3. 14 patients (42%) developed grade 1 or 2 neurologic toxicity (NTX), and 1 patient (3%) had reversible grade 4 NTX. The ORR was 85% with 15 patients (45%) achieving complete remission (CR), but 6 of them (40%) relapsed later. The median PFS was 11.8 months. All 16 evaluable patients who had ≥ PR had MRD-negative status. CAR T-cells were detected up to 1 year after the infusion. A phase 2 clinical trial (NCT03361748) for bb2121 is currently ongoing. bb21217 is an improved version of bb2121 with added phosphoinositide 3-kinase (PI3K) inhibitor to enrich memory-like T-cells. A multi-center phase 1 clinical trial of bb21217 (CRB-402; NCT03274219) has enrolled 22 RRMM patients who received a median of 7 previous lines of therapy including PIs, IMiDs and daratumumab, 18 patients had prior ASCT, 11 patients had ≥50% BCMA expression on MM cells by immunohistochemistry and 7 patients had high-risk cytogenetics [37]. bb21217 was well tolerated and toxicities were manageable. The response was evaluated in 18 patients with ≥2 months of follow up or PD within 2 months. Fifteen patients (83%) demonstrated clinical response, but 6 of them subsequently progressed. PFS in patients receiving bb2121 is not as long as some other BCMA CAR T-cells, and its efficacy needs to be investigated in previously heavily treated RRMM patients.

LCAR-B38M is a dual BCMA epitope-binding CAR T-cell therapy that directs against two distinct BCMA epitopes. The bi-epitope BCMA-binding moieties confer higher affinity to BCMA than other BCMA CAR constructs. LCAR-B38M uses 4-1BB as the costimulatory domain. BCMA expression was not required for enrolment in the trial. A phase 1 study of LCAR-B38M in 57 RRMM patients has been reported [38]. All the patients had ≥ grade 1 AEs with 37 of 57 patients (65%) had ≥ grade 3 AEs, including leukopenia, thrombocytopenia, and aspartate aminotransferase increase. Fifty one of Fifty seven patients (90%) had CRS; 4 patients (7%) at ≥ grade 3. The ORR was 88% with 39 (68%) CR, 3 (5%) VGPR, and 8 (14%) PR. MRD was negative for 36 patients (63%). The median time to response was 1 month. With a median follow-up of 8 months, the median PFS was 15 months and median OS was not reached. Another phase 1 study of LCAR-B38M enrolled 17 RRMM patients [39]. Ten patients experienced a mild CRS, 6 had severe but manageable CRS, and 1 died of very severe toxic reaction. ORR was 88.2% (15/17) with 13 stringent CR (sCR, 76.5%) and 2 VGPR. With a median follow-up of 417 days, 8 patients remained in sCR or VGPR, whereas 6 relapsed after sCR and 1 had PD after VGPR. CAR T-cells were high in most cases with stable response but low in 6 out of 7 relapse or PD cases. At median follow-up of 22 months, 6 patients (38%) remained progression-free [40]. The median PFS was 12 months in all patients, while the median PFS in MRD-negative patients with CR was 18 months. The median OS has not been reached. A phase 2 trial of LCAR-B38M in RRMM patients is ongoing (NCT03758417).

CT103A is a novel BCMA CAR-T with all sequences from human. A single-center and single-arm trial of CT103A (ChiCTR1800018137) was reported in 16 RRMM patients who received at least three previous lines of therapy including PIs and IMiDs or were refractory to both drugs, with the median follow-up of 195 days [41]. All patients (100%) had CRS with 10 patients at grade 1 or 2, 5 patients at grade 3 and 1 patient at grade 4. No NTX was observed. One patient died of lung infection 19 days after infusion. ORR was 100% (16/16) with 6 CR/sCR and 2 VGPR. Four patients who had participated in a prior CAR-T trial achieved CR/sCR, with 3 sCR and 1 VGPR. All 15 evaluable patients had MRD-negative status. CT103A in the peripheral blood peaked at 14 days and remained detectable in 12 patients at the last evaluation, indicating the rapid expansion and persistence of CT103A. With manageable safety profile, 100% ORR and favorable pharmacokinetics, CT103A is a promising treatment in RRMM patients.

Descartes-08 is autologous CD8^+^ T-cells transfected with mRNA-generated anti-BCMA CAR [42]. Descartes-08 induced cytotoxic degranulation and produced cytokine IFNγ, TNFαand IL-2 to kills MM cell lines and primary MM cells from both NDMM and RRMM patients. Descartes-08 also suppressed MM in a mouse model for disseminated human MM. Descartes-08 has a temporal limit in activity putative lower risk of severe CNS toxicities. A phase I trial (NCT03448978) for Descartes-08 is ongoing.

##### Allogeneic BCMA CAR T-cells

BCMA 1-R2 CAR T-cells were anti-BCMA allogeneic CAR T-cells from healthy donors in an attempt to eliminate the lag of treatment due to cell collection and manufacturing required for autologous CAR T-cells [43]. In BCMA 1-R2 CAR T-cells, the TRAC gene was eliminated by transcription activator-like effector nucleases (TALEN)-knockout strategy to reduce the potential of graft-versus-host disease (GvHD). The CD52 gene was also knocked out by TALEN to render BCMA 1-R2 CAR T-cells resistant to lymphodepletion therapy using anti-CD52 Ab, such as alemtuzumab. Furthermore, BCMA 1-R2 CAR T-cells were incorporated an intra-CAR rituximab binding domain as an off switch, thus allowing the elimination of these CAR T-cells by rituximab. When supplemented with human cytokine IL-7 and IL-15, BCMA 1-R2 CAR T-cells induced potent and persistent anti-tumor activity in MM mouse models. BCMA 1-R2 CAR-Ts were the safe “off the shelf” CAR T-cells and preserved their phenotype and potency after scale-up manufacturing, supporting a promising clinical evaluation in progressive MM patients.

#### Dual-antigen targeting CAR T-cell strategies

Dual-antigen targeting CAR T-cell strategies increase targetable MM cell antigens and reduce the risk of antigen-negative disease escape. For MM treatment, different strategies have been developed for CAR T-cells to target dual antigens including BCMA, such as dual CAR T-cell combination infusion of anti-CD19 and anti-BCMA CAR T-cells, BCMA-CS1 compound CAR (cCAR) T-cells targeting both BCMA and CS1, BCMA and CD19 bispecific CAR-T, and APRIL-CAR T-cells targeting both BCMA and TACI.

##### Dual CAR T-cell combination infusion of anti-CD19 and anti-BCMA CAR T-cells

Similar to fully differentiated plasma cells, most myeloma cells are CD19-negative. A small subset of CD19-positive myeloma cells that are less differentiated have drug resistant and disease-promoting qualities. Low expression of CD19 appears to be more common on MM cells than previously thought, and correlated with poor survival [44]. Anti-CD19 CAR T-cell therapy has shown activity in some of MM patients with premature clones [45].

In a single-centre, single-arm, phase 2 clinical trial (ChiCTR-OIC-17011272), BCMA CAR T-cells were infused in combination with CD19 CAR-T cells in 21 RRMM patients [46]. At a median follow-up of 179 days, ORR was 95% (20/21), including 43% (9/21) sCR, 14% (3/21) CR, 24% (5/21) VGPR, and 14% (3/21) PR. The most common AE was CRS (90%, 19/21), in which 18 patients (86%) presented grade 1–2 CRS. The most common SAE was hematological toxicities (20/21 patients). Common grade 3 or higher AEs included neutropenia, anemia, and thrombocytopenia. One patient died of hemorrhagic stroke, likely due to sustained thrombocytopenia. No deaths were judged to be treatment-related. Long-term follow-up is needed to evaluate the extended activity and safety of this dual CAR T-cell therapy.

##### BCMA bispecific CAR-T

BCMA-CD38 bispecific CAR-T is bispecific CAR T-cells targeting both BCMA and CD38, incorporating the anti-CD38 and anti-BCMA scFv in tandem plus 4-1BB signaling and CD3ζ domains [47]. A phase 1, dose-escalation trial of BCMA-CD38 bispecific CAR-T (ChiCTR1800018143) has been conducted in 16 RRMM patients who received at least two previous lines of therapy including PIs and IMiDs, with 10 genetic abnormalities and 5 extramedullary lesions. The median follow-up was 36 weeks. Ten patients (10/16, 62.5%) had CRS (4 at grade ≥ 3), all manageable. In almost all the patients, hematological toxicities were reduced in the first month after infusion. No NTX was observed. ORR was 87.5% (14/16) with 8 sCRs, 2 VGPRs and 4 PRs. Fourteen patients (87.5%) became MRD-negative. In 8 sCR patients, 5 patients maintained sCR, 2 transformed to VGPR and 1 to PR, with the longest duration of sCR over 51 weeks. The peak expansion time of the CAR T-cells in sCR patients was the 2nd week after infusion, and was earlier than patients not achieving sCR. Extramedullary lesions were eliminated in all 5 patients (100%). 9-month PFS rate was 75%. The median PFS had not been reached. The efficacy and toxicity of these novel bispecific CAR-T are generally comparable to monospecific anti-BCMA CAR T and dual CAR T-cell combination infusion. These results supported further development of bispecific CAR-T in RRMM patients.

BCMA-CD19 bispecific CAR-T is another bispecific CAR T-cells targeting both BCMA and CD19. It is constructed by linking BCMA and CD19 scFv, a CD8 hinge, transmembrane domain, co-stimulatory domain and CD3ζ [48]. The BCMA-CD19 bispecific CAR-T was manufactured in the FasT CAR-T platform with shortened production time of 1 day. BCMA-CD19 bispecific CAR-T potently killed CD19^+^ and/or BCMA^+^ MM cell lines and effectively eliminated tumor in MM xenograft mice, with more potent cytotoxicity than single CAR-T. At a median follow-up of 68 days, all 5 MM patients enrolled responded to BCMA-CD19 bispecific CAR-T, including 1 sCR, 3 VGPR, and 1 PR. Three patients experienced grade 1 CRS and no SAE was observed. A clinical trial of FasT BCMA-CD19 CAR-T is ongoing.

##### BCMA-CS1 cCAR T-cells

BCMA-CS1 cCAR (BC1cCAR) T-cells are comprised of a complete BCMA-CAR linked to a complete CS1-CAR via a self-cleaving P2A peptide, which expresses both BCMA and CS1 CAR molecules on the T-cell surface [49]. CS1, which is also referred to as CD319 or SLAMF7, is specifically expressed at high level in normal plasma cells and myeloma cells, and play an important role in myeloma parthenogenesis. Elotuzumab targeting CS1 has been successfully used in the treatment of MM, and CS1-CAR therapy has demonstrated promising efficacy for MM in pre-clinical studies [50, 51]. BC1cCAR T-cells possess consistent, potent and direct cytotoxicity against each target antigen BCMA or CS1 MM cells. BC1cCAR T-cells produced higher cytotoxicity against MM populations than single-expressing CAR T-cells in mouse models, including MM1S cell line, BCMA-K562, CS1-K562, and mixed BCMAK562/CS1-K562.

##### APRIL-CAR T-cells

APRIL is the natural ligand for BCMA and TACI. Both BCMA and TACI are expressed in majority of primary MM samples. Lee et al. reported that all MM cases expressed BCMA and 78% (39/50) of them also expressed TACI [52]. In normal tissues, TACI expression is restricted to the lymphoid compartment with distribution pattern similar to that of BCMA. Schmidts et al. found that 27 and 28 of 29 MM patients expressed BCMA and TACI, respectively, with expression level of ≥20% of total plasma cells [53].

APRIL-CAR (ACAR) T-cells are the third-generation CAR using a truncated form of monomeric APRIL as the tumor-targeting domain, and recognizes both BCMA and TACI on MM cells [52]. ACAR T-cells induced significant cytolytic activity even at low antigen levels in myeloma cells. Elimination of MM cells was also observed in an intramedullary mouse myeloma model. In mouse models of tumor escape, ACAR T-cells eradiated both BCMA+TACI^−^ and BCMA-TACI^+^ cells, whereas BCMA-CAR T-cells alone resulted in outgrowth of BCMA-negative tumors. Therefore, in comparison with targeting BCMA, ACAR dual-antigen targeting of BCMA and TACI facilitates sustained MM suppression in the event of BCMA down-regulation or loss in MM patients who express both antigens. Tumor killing by ACAR T-cells was unaffected by physiological levels of soluble APRIL or TACI, but attenuated at the highest level of sBCMA (1000 ng/ml). A phase 1/2 clinical trial of APRIL-CAR T-cells is ongoing (NCT03287804).

TriPRIL CARTs contain multimeric APRIL binding domain with 3 truncated APRIL monomers, and preserve natural trimeric conformation of APRIL [53]. TriPRIL CARTs induced activity against both BCMA^+^ and BCMA^−^ MM cell lines and MM xenograft mouse models, as well as primary MM patient-derived myeloma cells. In addition to killing BCMA^+^ MM cells, TriPRIL CARTs also eliminated BCMA-TACI^+^ MM cells. TriPRIL CARTs were also superior to monomeric APRIL CARTs in affinity, long-term proliferative capacity and polyfunctionality of T-cells. TriPRIL CARTs are well designed with all the sequences from human and promising in the treatment of antigen escape from monospecific target therapy.

#### Other BCMA CAR cells

Major obstacles in clinical use of CAR T-cells are high risk of CRS and NTX. Expressing CAR on other cells have been proposed. Invariant natural killer T-cells (iNKTs) express a monomorphic TCR and do not cause graft versus host disease (GVHD). BCMA-iNKT-CAR was potent against MM cells in MM cell lines and MM mouse model. Since iNKTs express IL-7R, NT-I7 (a long-acting IL-7) enhanced BCMA-iNKT-CAR antitumor efficacy and prolonged iNKT-CAR cell survival in vivo [54].

## Conclusion

Despite the clinical use of PIs, IMiDs, and MoAbs in the past decade, MM remained incurable. To achieve a cure, effective targeted immunotherapies need to be developed to eradicate non-proliferating MPCs, sustain deep MRD negativity, and normalize anti-MM immune system. BCMA, a specific antigen for anti-MM therapy, has been successfully targeted by immunotherapeutic strategies, especially BCMA-peptide specific CTL vaccines, ADC, BsAbs, and BCMA-targeted CAR T-cells.

Another important issue is to choose appropriate anti-BCMA immunotherapy for individual patients. The decision-making must consider multiple factors, including biological features of MM, comorbidities, prior therapies and toxicities, economic conditions, access to the novel immunotherapy, the patient’s preference, and the balance of benefits and side effects from the therapy. Among these BCMA-targeting immune strategies, BCMA-peptide specific CTL vaccines have achieved memory anti-MM responses and induce epitope spreading to sustain anti-MM efficacy [55, 56]. Vaccines may also be combined with inhibitors of immune checkpoint to enhance anti-MM cellular immunity in MM patients. ADC can be used in immune-compromised RRMM patients. Anti-BCMA ADC could be conjugated to drugs to expand the target cells to non-proliferating MPCs [23]. Anti-BCMA BsAbs and CAR T-cells enhance anti-MM immunity in MM patients. Compared with CAR T-cells, anti-BCMA BsAbs are off-the-shelf commercial products with potent T-cell-mediated killing of specific BCMA^+^ MM cells without requirement ex vivo engineering and T-cell expansion. BsAbs may have potential disadvantages in MM patients with bulky extramedullary lesions and CNS involvement [57]. BCMA-targeted CAR cells induce deep response in RRMM, and could eliminate bulky extramedullary lesions, but the responses are often temporary. The main reasons for the relapse are the down-regulation or loss of BCMA expression on the MM cell surface and CAR T-cell exhaustion, which could be the result of CAR T-cell induced trogocytosis or the suppressive myeloid cells in the myeloma niche [58–60]. Dual-antigen targeting CAR T-cell strategies reduce the risk of BCMA escape and increase the targeting antigen of MM cells. Recent studies indicated that γ-secretase inhibitors could decrease sBCMA while increasing surface BCMA expression in short-term administration, thus augment the antitumor activity of BCMA CAR T-cell therapy [61]. CAR-NKs are universal, cheap and fast “off-the-shelf” cellular therapies, and need to be taken into consideration in anti-BCMA CAR-cell strategies.

Immunotherapeutic strategies targeting BCMA need to be improved significantly to eradicate MM cells permanently. Combination of different immunotherapeutic strategies targeting BCMA or multi-target immune therapeutic strategies (such as multi-antigen targeting CAR cells and NanoMuTEs), together with immune modulatory agents (such as IMiDs and check point inhibitors) to normalize anti-MM immune system in MRD negative patients, may be required to achieve such a goal.

## Data Availability

Not applicable.
